# Three generations of epigenetic clocks in mediating the adverse effect of smoking on metabolic health

**DOI:** 10.1080/17501911.2025.2494497

**Published:** 2025-04-18

**Authors:** Chen-Yu Yeh, Wan-Yu Lin

**Affiliations:** a Institute of Epidemiology and Preventive Medicine, College of Public Health, National Taiwan University, Taipei, Taiwan; b Institute of Health Data Analytics and Statistics, College of Public Health, National Taiwan University, Taipei, Taiwan; c Master of Public Health Program, College of Public Health, National Taiwan University, Taipei, Taiwan

**Keywords:** Biological age, DNA methylation, epigenetic age acceleration, insulin resistance, Taiwan Biobank

## Abstract

**Aims:**

Metabolic syndrome (MetS) is a composite disorder that includes abdominal obesity, impaired glucose levels, high blood pressure, and dyslipidemia. Smoking can alter epigenetic profiles and is a critical modifiable risk factor for MetS. We aim to explore the epigenetic age acceleration (EAA) that can mainly deliver smoking influences on metabolic health.

**Methods:**

We conducted a mediation analysis of 2,474 individuals with data in the Taiwan Biobank. Current and former smoking and the respective pack-years were included as four exposure factors. Seven markers of DNA methylation (DNAm) covering three generations of epigenetic clocks were included as mediators. Seven metabolic outcomes included MetS status (yes vs. no) and six related traits.

**Results:**

GrimEAA and DunedinPACE mediated the associations of the four smoking factors with MetS, fasting glucose, triglyceride, and high-density lipoprotein cholesterol levels (false discovery rate < 0.05). GrimEAA and DunedinPACE respectively mediated 48.2% and 24.2% of current smoking’s effect on MetS and 60.9% and 26.1% of current smoking pack-year’s effect on MetS risk. The DNAm plasminogen activator inhibitor 1 level mediated the adverse effects of current smoking status and pack-years on all seven metabolic outcomes.

**Conclusion:**

The GrimEAA-mediated proportions were approximately two times greater than the DunedinPACE-mediated proportions.

## 1. Introduction

Metabolic syndrome (MetS) is prevalent worldwide. For example, approximately 40% of 60-year-old U.S. adults and 37% of Taiwanese adults aged above 40 years have MetS, causing a substantial economic burden [1] (Taiwan’s data reported from https://www.hpa.gov.tw/EngPages/Detail.aspx?nodeid=1076&pid=17250). Fortunately, MetS can be prevented in an early stage. Smoking is a leading modifiable risk factor for MetS. A close relationship between smoking and MetS has been reported. According to a meta-analysis of 13 studies involving 56,691 individuals and 8,688 cases of MetS, active smoking is associated with an increased risk of MetS (relative risk = 1.26) [2]. Moreover, a Korean study consisting of 808 adults under 40 years of age revealed that smokers had a 2.4-fold greater risk of MetS than nonsmokers [3].

MetS increases the risk of stroke, diabetes, heart disease, and several critical health issues. According to the definition from the National Cholesterol Education Program Adult Treatment Panel III, MetS is diagnosed if an individual has three or more of the following five conditions: (1) a waist circumference >40 inches (men) or 35 inches (women); (2) a blood pressure > 130/85 mmHg; (3) a fasting triglyceride level >150 mg/dl; (4) a fasting high-density lipoprotein cholesterol (HDL-C) level <40 mg/dl (men) or 50 mg/dl (women); and (5) a fasting glucose (FG) level >100 mg/dl [4].

Cigarette smoking was found to be responsible for reduced insulin sensitivity and insulin resistance, worsening the control of blood glucose and leading to diabetes [5]. MetS is also called “insulin resistance syndrome.” Reduced insulin sensitivity increases the risk of diabetes, abdominal obesity [6], dyslipidemia [6], hypertension [7], and cardiovascular diseases [8]. Smoking can globally change DNA methylation (DNAm) levels [9,10]. The effect of smoking on MetS may occur through epigenetic age acceleration (EAA), an aggregation of information from hundreds of aging-related cytosine-phosphate-guanine (CpG) sites.

On the other hand, the relationship between smoking and abdominal obesity is complicated. Nicotine can suppress appetite. Weight gain commonly occurs after smoking cessation [9]. Animal studies demonstrated that nicotine effectively decreased abdominal fat in mice [11]. However, a recent Mendelian randomization study revealed a positive causal effect of smoking on waist-hip ratio [12]. The debate about whether smoking may reduce obesity risk is ongoing. Although mediation analysis cannot definitively refer to causation, investigating mediators of the relationship between smoking and abdominal obesity may help to address this issue.

In addition to the relationship with abdominal obesity, the relationship between blood pressure and cigarette smoking remains equivocal. Smoking can cause an acute increase in blood pressure [13]. Smoking at least two cigarettes hourly for eight hours induces a continuous rise in blood pressure [14]. Surprisingly, epidemiological studies have reported lower blood pressure levels in smokers than in nonsmokers [15,16]. Common explanations include a failure to account for critical covariates. As a consequence, whether cigarette smoking is a risk factor for hypertension is still unknown [16]. Uncovering the mediators that link smoking with blood pressure may facilitate our understanding of this topic.

With the advancement of epigenetic studies, smoking has been found to alter DNAm levels at many CpG sites [9] and to deteriorate metabolic health [17,18]. Instead of investigating individual CpG sites that may suffer from a harsh penalty of multiple testing, we focused on EAA, which aggregates the information from numerous aging-related CpG sites. We aimed to explore the epigenetic clock that mainly delivers smoking influences on metabolic health. Our previous work revealed that the associations between smoking and diabetes-related outcomes (FG and glycated hemoglobin [HbA1c] levels) are partially mediated by the CpG levels of the second- and third-generation epigenetic clocks [19]. In this work, we aimed to explore the role of EAA as a mediator between smoking and MetS risk or MetS-related traits. This investigation assessed the role of EAA in mediating the effects of smoking on MetS risk, abdominal obesity, dyslipidemia, and hypertension.

## 2. Materials and methods

### 2.1. Study subjects from the Taiwan Biobank

The Taiwan Biobank (TWB) has included ~ 200,000 community volunteers from the population of Taiwanese residents aged 30 to 70 years without a previous cancer diagnosis since October 2012. Each participant signs written informed consent, completes a lifestyle questionnaire administered by TWB health professionals, undergoes a physical examination, and provides blood and urine samples after fasting for at least 6 hours. According to the county population size and male-female ratio, 2,474 of the ~ 200,000 individuals were randomly selected for DNAm quantification from 2016–2021.

TWB was approved by the Ethics and Governance Council of Taiwan Biobank and the Institutional Review Board on Biomedical Science Research/IRB-BM, Academia Sinica, Taiwan. Each participant completed written informed consent following the principles of the Declaration of Helsinki and institutional requirements. TWB approved our application to analyze the data on 18 February 2020, with the application number TWBR10810–07. The current study further received approval from the Research Ethics Committee of the National Taiwan University Hospital with the number NTUH-REC no. 201805050RINB.

### 2.2. Smoking factors (X: exposure)

The four smoking factors included (1) current smoking (over a six-month duration); (2) smoking pack-years for current smokers; (3) former smoking (quitting smoking for at least six months); and (4) smoking pack-years for former smokers.

### 2.3. Metabolic outcomes (Y: outcome)

We investigated whether EAA mediates the associations between the four smoking factors and MetS together with its six related traits. The criteria for MetS are based on the recommendations of Taiwan’s Ministry of Health and Welfare. MetS is defined as the presence of at least three of the following five problems: (1) abdominal obesity with a waist circumference ≥90 cm (~35 inches) for men and ≥80 cm (~31 inches) for women; (2) a high blood pressure with a systolic blood pressure (SBP) ≥ 130 mmHg or a diastolic blood pressure (DBP) ≥ 85 mmHg; (3) an FG level ≥100 mg/dL; (4) a fasting triglyceride level ≥150 mg/dL; and (5) an HDL-C level <40 mg/dL for men and <50 mg/dL for women. In total, we studied **seven** health outcomes: MetS (dichotomous, yes vs. no), waist circumference, SBP, DBP, FG level, triglyceride level, and HDL-C level. Except for MetS, all traits were measured on a continuous scale.

### 2.4. Epigenetic age acceleration (M: mediator)

The blood DNAm levels of 2,474 individuals were quantified with the Illumina Infinium MethylationEPIC BeadChip, which covers approximately 860,000 CpG sites. The quality control and normalization processes for the DNAm data were documented in our previous work [20]. We used the online DNAm Age Calculator from Horvath’s laboratory, available at https://dnamage.genetics.ucla.edu/new, to calculate four measures of EAA and two components of GrimEAA, including HannumEAA [21], intrinsic epigenetic age acceleration (IEAA) [22], PhenoEAA [23], GrimEAA [24,25], DNAm-based smoking pack-years (DNAmPACKYRS) [24,25], and DNAm plasminogen activator inhibitor 1 levels (DNAmPAI1) [24,25]. DunedinPACE [26] was computed by the R package DunedinPACE (https://github.com/danbelsky/DunedinPACE). In summary, seven epigenetic markers, in turn, were included as mediators.

We further excluded 7, 1, 2, 5, 54, 0, and 1 extreme outliers for HannumEAA, IEAA, PhenoEAA, GrimEAA, DNAmPACKYRS, DNAmPAI1, and DunedinPACE. Because some individuals were heavy smokers, we detected 54 extreme outliers from the right-skewed DNAmPACKYRS distribution. Extreme outliers were defined as values larger than $Q_{3}+3\times \left(Q_{3}-Q_{1}\right)$ or smaller than $Q_{1}-3\times \left(Q_{3}-Q_{1}\right)$, in which $Q_{1}$ and $Q_{3}$ represent the first and third quartiles, respectively.

### 2.5. Statistical analyses

Statistical analyses were performed using R software (version 4.3.2). Mediation analysis was conducted with the ‘mediation’ R package [27]. When the outcome was continuous, we performed a *z*-score transformation on the trait to facilitate a fair comparison of the mediation effects on various outcomes. More details can be found in the Supplementary Method of the Supplementary Materials.

With the function “mediate,” we used the non-parametric bootstrapping method to estimate the 95% CI of the mediation effects (i.e., boot = TRUE), given that the number of simulations was 2000 (sims = 2000). After considering four smoking factors, seven traits, and seven epigenetic markers, we obtained 196 p-values from the 196 ( = 4 × 7 × 7) mediation analyses. The Benjamini-Hochberg false discovery rate (FDR) control method [28] was then applied to correct for multiple comparisons.

## 3. Results

### 3.1. Smoking-outcome associations

Table 1 shows the primary characteristics of the 2,474 TWB participants categorized according to their smoking status. Among the 2,474 individuals, 1,879 (76.0%) were nonsmokers, 312 (12.6%) were former smokers, and 283 (11.4%) were current smokers. The average number of cigarettes per day was 18.8 (sd = 14.3) for former smokers and 13.7 (sd = 10.4) for current smokers. Over 80% of the current or former smokers were males, whereas 60.8% of the nonsmokers were females. A total of 16.3% ( = 306/1879) of nonsmokers, 26.3% ( = 82/312) of former smokers, and 31.1% ( = 88/283) of current smokers had MetS according to the MetS definition from Taiwan’s Ministry of Health and Welfare. As shown by Supplementary Table S1, the percentage of MetS increases with smoking intensity. Furthermore, given similar smoking intensity, current smokers have a higher risk of MetS compared with former smokers.

**Table 1.** Characteristics of the 2474 individuals stratified by smoking status.

	Non-smokers	Former smokers^a^	Current smokers^b^	*p*-value^c^
Total	1879 (76.0%)	312 (12.6%)	283 (11.4%)	
Age range	30–70	30–70	30–70	
Age (year)	49.4 (11.2)	52.9 (10.2)	48.8 (10.6)	3.93E–07
Sex				3.76E–84
Males	736 (39.2%)	272 (87.2%)	235 (83.0%)	
Females	1143 (60.8%)	40 (12.8%)	48 (17.0%)	
BMI (kg/m^2^)	24.1 (3.7)	25.3 (3.2)	25.4 (3.7)	1.32E–14
Metabolic syndrome	306 (16.3%)	82 (26.3%)	88 (31.1%)	1.01E–10
Waist circumference (cm)	83.0 (10.1)	88.4 (9.0)	87.8 (9.9)	7.55E–29
Systolic blood pressure (mmHg)	116.1 (17.3)	123.7 (16.8)	120.2 (16.6)	3.36E–14
Diastolic blood pressure (mmHg)	72.2 (11.1)	77.5 (10.3)	75.3 (11.4)	2.51E–17
Fasting glucose (mg/dl)	94.4 (16.5)	98.9 (20.3)	102.2 (34)	7.98E–16
Triglyceride (mg/dl)	110.8 (101.5)	123.2 (81.7)	160.8 (144.3)	4.79E–19
HDL-C (mg/dl)	55.6 (13.7)	49.7 (12.0)	46.2 (11.7)	4.71E–36
Educational attainment^d^	5.6 (0.9)	5.6 (0.9)	5.4 (0.8)	0.0002
Regular exercise^e^	819 (43.6%)	178 (57.1%)	95 (33.6%)	2.98E–08
Smokers’ pack-years				
Former smokers	–	12.9 (14.1)	–	
Current smokers	–	–	20.8 (19.9)	
Number of cigarettes per day				
Former smokers		18.8 (14.3)		
Current smokers			13.7 (10.4)	
Drinking^f^	72 (3.8%)	30 (9.6%)	69 (24.4%)	1.16E–36

The data are presented as the n (%) or mean ± standard deviation (sd).

^a^Former smokers were defined as those who “had smoked for at least six months but had quit smoking for at least six months at the time of joining the TWB study.”

^b^Current smokers were defined as those who “had smoked for at least six months and had not quit smoking at the time of joining the TWB study.”

^c^The p-value of testing the mean or proportion difference among the three smoking groups was based on a Kruskal-Wallis test for continuous factors or a Chi-square test for categorical factors.

^d^Educational attainment was an integer ranging from 1 to 7: 1 “illiterate,” 2 “no formal education but literate,” 3 “primary school graduate,” 4 “junior high school graduate,” 5 “senior high school graduate,” 6 “college graduate,” and 7 “Master’s degree or higher.”

^e^Regular exercise was defined as “engaging in exercise at least thrice a week, with a duration >30 minutes each time.” Exercise included leisure-time activities such as badminton, tennis, swimming, yoga, jogging, cycling, mountain climbing, dancing, etc.

^f^Drinking was defined as “having consumed 150 c.c. of alcoholic beverages per week for at least six months.”

Concerning individual traits, we found that FG and triglyceride levels followed a similar trend, i.e., nonsmokers’ levels < former smokers’ levels < current smokers’ levels. In contrast, HDL-C levels showed the opposite trend, i.e., nonsmokers’ levels > former smokers’ levels > current smokers’ levels.

On the other hand, waist circumference, SBP, and DBP presented the following trend: former smokers’ values > current smokers’ values > nonsmokers’ values. Because former smokers (mean age = 52.9 years) were older than current smokers (mean age = 48.8 years), this pattern could be confounded by chronological age or other covariates. To control for these factors, we regressed metabolic outcomes (Y) on smoking factors (X) while adjusting for covariates, i.e., chronological age, sex, educational attainment, BMI, regular exercise status, drinking status, and the proportions of five cell types (B lymphocytes, CD4+ T cells, CD8+ T cells, monocytes, and natural killer cells).

The top parts of Supplementary Tables S2-S5 show the total effect of the four smoking variables (X) on the seven metabolic outcomes (Y). We used p-values to evaluate whether the 28 X-Y (4 Xs and 7 Ys) associations were significant. Because X-Y associations were not our primary purpose in this work, we did not adjust the p-values according to multiple tests at this stage. P-values less than 0.05 were considered to indicate statistical significance.

Current and former smoking were associated with a 0.0993 (95% CI = 0.0474 ~ 0.1535, Supplementary Table S2) and 0.0534 (95% CI = 0.0087 ~ 0.0992, Supplementary Table S4) increased probability of having MetS, respectively. Each current and former smoking pack-year was associated with a 0.0023 (95% CI = 0.0010 ~ 0.0037, Supplementary Table S3) and 0.0023 (95% CI = 0.0004 ~ 0.0041, Supplementary Table S5) increased probability of having MetS, respectively.

We further explored individual traits. Consistent with the descriptive statistics (Table 1), current smoking and pack-years were associated with increased FG and triglyceride levels but were negatively associated with HDL-C levels (Supplementary Tables S2-S3). Former smoking and pack-years were associated with increased waist circumference (Supplementary Tables S4-S5).

### 3.2. Smoking-outcome associations mediated by epigenetic markers

The first-generation epigenetic clocks (HannumEAA and IEAA) demonstrated no significant mediation effects on any of the smoking-metabolism associations (FDR >0.05, Supplementary Tables S2-S5). GrimEAA and DunedinPACE mediated the associations of the four smoking factors with MetS, FG, triglyceride, and HDL-C levels (FDR <0.05, Supplementary Tables S2-S5). GrimEAA and DunedinPACE respectively mediated 48.2% and 24.2% of current smoking’s effect on MetS risk (Supplementary Table S2); 60.9% and 26.1% of current smoking pack-years effect on MetS risk (Supplementary Table S3); 21.7% and 9.4% of former smoking’s impact on MetS risk (Supplementary Table S4); 30.4% and 13.0% of former smoking pack-years effect on MetS risk (Supplementary Table S5). The GrimEAA-mediated proportions were approximately two times greater than the DunedinPACE-mediated proportions.

**Figure 1.**
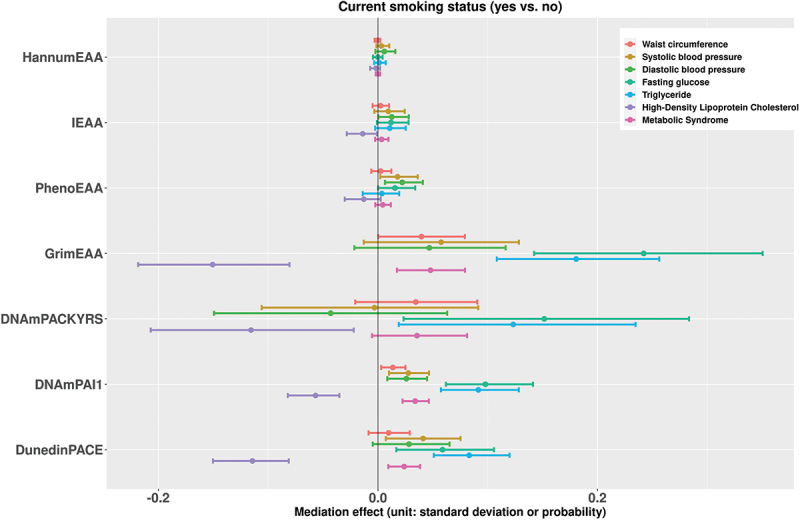
The estimates of the indirect effects (95% confidence intervals) of current smoking status on the seven metabolic outcomes associated with DNAm markers.

**Figure 2.**
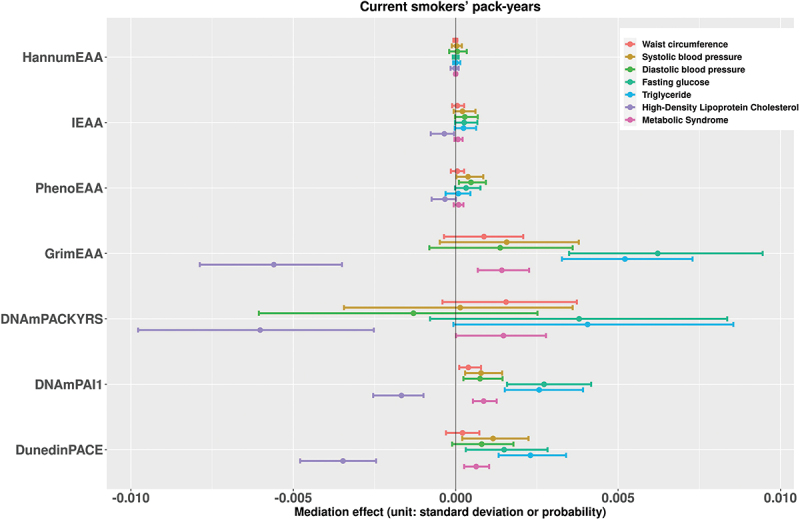
The estimates of the indirect effects (95% confidence intervals) of current smoking pack-years on the seven metabolic outcomes associated with DNAm markers.

**Figure 3.**
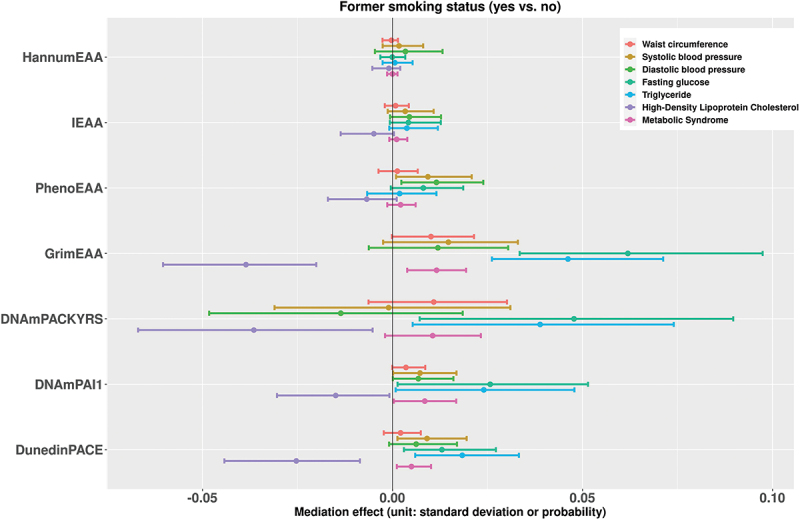
The estimates of the indirect effects (95% confidence intervals) of former smoking status on the seven metabolic outcomes associated with DNAm markers.

**Figure 4.**
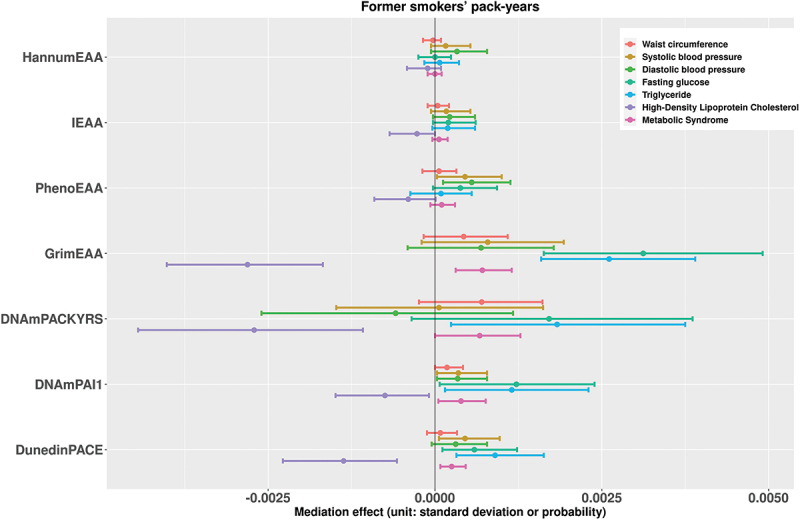
The estimates of the indirect effects (95% confidence intervals) of former smoking pack-years on the seven metabolic outcomes associated with DNAm markers.

Figure 1 presents the estimates of the mediation effects of current smoking status on the seven metabolic outcomes associated with DNAm markers. Figure 2 shows the results when the exposure is changed to the current smoking pack-years. We also put the results for former smoking status and former smoking pack-years in Figures 3 and 4, respectively. With respect to individual traits of MetS, the mediating effects of GrimEAA and DunedinPACE on the associations between the four smoking factors and FG and triglyceride levels were positive. This finding indicated that former and current smoking both increased FG and triglyceride levels through the CpG sites of GrimEAA and DunedinPACE. In contrast, former and current smoking decreased HDL-C levels through the CpG sites of these two epigenetic markers (Supplementary Tables S2-S5; Figures 1–4).

Before mediation analysis, we performed z-score transformation on the waist circumference, systolic blood pressure, diastolic blood pressure, fasting glucose level, triglyceride level, and high-density lipoprotein cholesterol level. Therefore, the unit of the mediation effects for these six continuous traits is the standard deviation. On the other hand, the unit of the mediating effect of “metabolic syndrome” is the probability.

Before mediation analysis, we performed z-score transformation on the waist circumference, systolic blood pressure, diastolic blood pressure, fasting glucose level, triglyceride level, and high-density lipoprotein cholesterol level. Therefore, the unit of the mediation effects for these six continuous traits is the standard deviation. On the other hand, the unit of the mediating effect of “metabolic syndrome” is the probability.

Before mediation analysis, we performed z-score transformation on the waist circumference, systolic blood pressure, diastolic blood pressure, fasting glucose level, triglyceride level, and high-density lipoprotein cholesterol level. Therefore, the unit of the mediation effects for these six continuous traits is the standard deviation. On the other hand, the unit of the mediating effect of “metabolic syndrome” is the probability.

Before mediation analysis, we performed z-score transformation on the waist circumference, systolic blood pressure, diastolic blood pressure, fasting glucose level, triglyceride level, and high-density lipoprotein cholesterol level. Therefore, the unit of the mediation effects for these six continuous traits is the standard deviation. On the other hand, the unit of the mediating effect of “metabolic syndrome” is the probability.

DNAmPAI1 mediated the associations of current smoking and the pack-years of current smokers with all seven health outcomes (FDR <0.05, Supplementary Tables S2-S3). Through DNAmPAI1, current smoking increased the probability of MetS, waist circumference, SBP, DBP, FG level, and triglyceride level but decreased the HDL-C level (Figures 1–2). HDL-C is the so-called “good cholesterol” because an elevated HDL-C level is usually linked to reduced cardiovascular risk. In summary, these seven significant mediating effects consistently indicate that smoking has harmful effects on human metabolic health.

PhenoEAA had a positive mediating effect on the associations between the four smoking factors and DBP (FDR <0.05, Supplementary Tables S2-S5). DNAmPACKYRS presented a negative mediating effect on the association between current or former smoking pack-years and the HDL-C level (FDR <0.05, Supplementary Tables S3 & S5).

In summary, all the significant mediating effects indicated that smoking is detrimental to metabolic health. Even after a period of no smoking for more than six months (definition of “former smoking” in the TWB data), the adverse effects of smoking still influence metabolism through EAA.

## 4. Discussion

Recently, MetS traits were shown to be associated with markers of epigenetic aging, especially DNAmPAI1 (one component of GrimAge) [29]. Moreover, previous studies have suggested that obesity is associated with epigenetic clock acceleration [30]. In the present study, our hypothesis was the other way around. According to the central dogma of life, DNA is transcribed into RNA, and RNA is translated into proteins. In this process, methyl groups can attach to DNA, affecting gene transcription. Because cigarette smoking is a well-known environmental factor that can globally change DNAm levels, we hypothesize that smoking deteriorates metabolism through various DNAm markers. The seven popular DNAm markers have attracted much attention and have been used to summarize epigenetic profiles. The first-, second-, and third-generation epigenetic clocks were included as mediators in this study.

Elevated plasminogen activator inhibitor 1 (PAI1) levels are a leading indicator of insulin resistance and MetS [31]. As an epigenetic surrogate marker of PAI1, DNAmPAI1 mediates the adverse effects of current smoking on all seven metabolic traits. However, after quitting smoking for at least six months, the role of DNAmPAI1 as a mediator became nonsignificant (FDR >0.05). This evidence highlights the shorter memory of cigarette smoking for the 211 CpG sites of DNAmPAI1 (211 CpG sites were among the 1,030 CpG sites of GrimEAA). In contrast, the 1,030 CpGs of GrimEAA and 173 CpGs of DunedinPACE had a more extended memory regarding smoking. These two EAA measures significantly mediated the effect of smoking (for both current and former smokers) on MetS and FG, triglyceride, and HDL-C levels (FDR <0.05).

While smoking temporarily increases blood pressure levels, paradoxically, extensive epidemiological studies have shown slightly lower blood pressure levels in smokers than in nonsmokers [15,16]. Compared with those of nonsmokers, the TWB data revealed slightly lower DBP and SBP values in current smokers and slightly higher DBP and SBP values in former smokers. Increased blood pressure has been observed during smoking cessation, although the mechanism remains unclear [32]. While considerable adverse effects of smoking on health have been well established, the role of smoking as a risk factor for hypertension has not been confirmed by epidemiological evidence. A possible explanation is the failure to control for critical confounders. Our results revealed that smoking is associated with elevated blood pressure through DNAm markers such as DNAmPAI1 and PhenoEAA.

EAA has been widely applied to predict biological aging rates [33]. DNAm is a vital molecular process in response to environmental changes, making it a plausible intermediate pathway linking environmental factors to phenotypes [34]. Recently, the role of DNAm in mediating environmental effects (e.g., smoking and adverse childhood experiences) on health outcomes has received much attention [35,36]. According to Klopack *et al*.’s study based on U.S. DNAm data, the second- and third-generation epigenetic clocks (PhenoEAA, GrimEAA, and DunedinPoAm38 [the earlier version of DunedinPACE]) can mediate the effects of smoking pack-years on high blood pressure, heart disease, cancer, and mortality [35].

Our work also investigated blood pressure traits, i.e., DBP and SBP. Similar to the results of Klopack *et al*. [35], we found that PhenoEAA had a positive mediating effect on the association between the four smoking factors and DBP (FDR <0.05). However, the mediating effects of GrimEAA and DunedinPACE were not significant (FDR >0.05). This inconsistency between the results of Klopack *et al*. [35] and those of our work may be partly due to the different strategies for handling multiple tests. While Klopack *et al*. [35] did not correct for multiple testing, we adjusted p-values according to the Benjamini-Hochberg FDR control method [28]. Without correction for multiple testing, DunedinPACE significantly mediated the effects of the four smoking factors on SBP (*p* < 0.05). Furthermore, the inconsistency between the two studies may be partly due to population differences. In Klopack *et al*.’s study [35], 73% of the individuals were non-Hispanic white, 12% were non-Hispanic black, 11% were Hispanic, and 4% were non-Hispanic other races. In contrast, over 99% of the TWB participants were Han Chinese, including Minnan Taiwanese, Hakka Taiwanese, and people of Chinese descent [37,38].

A Korean study published in 2020 revealed that GrimEAA and DNAmPAI1 were associated with MetS in middle-aged adults [39]. Because that study was conducted before 2022, DunedinPACE [26] was not investigated. This work extends the EAA-MetS relationship to the smoking-EAA-MetS association link, as smoking can globally change DNAm levels and EAA. In addition, we considered the third-generation epigenetic clock DunedinPACE. Our results revealed that DunedinPACE mediated the effects of smoking on MetS and FG, triglyceride, and HDL-C levels. The role of DunedinPACE as a mediator was similar to that of GrimEAA. Nonetheless, the mediating effects of DunedinPACE were only approximately half those of GrimEAA.

This work has two main limitations. First, our analysis could not imply causation because the TWB data were collected cross-sectionally. The data on metabolic health outcomes and DNAm levels were collected from TWB participants at a single time point. Second, our analysis results relied on self-reported smoking status without biological confirmation. Although self-reported smoking status is commonly used in research [40,41], it should be noted as a weakness.

## 5. Conclusion

Regarding the three generations of epigenetic clocks in mediating the adverse effect of smoking on metabolic health, the GrimEAA-mediated proportions were approximately two times greater than the DunedinPACE-mediated proportions. One component of GrimEAA, DNAmPAI1, mediated the effect of current smoking on all seven metabolic outcomes. Smoking was indirectly associated with increased waist circumference, SBP, DBP, FG levels, triglyceride levels, and MetS risk but reduced HDL-C levels through DNAmPAI1. All these indirect impacts are detrimental to metabolic health. It is widely known that smoking can globally change DNAm levels [9,10]. Moreover, EAA is associated with MetS, as reported by several studies [29,42]. However, this work is the first study to evaluate the three generations of epigenetic clocks in mediating the adverse effect of smoking on metabolic health.

## Supplementary Material

Supplemental Material

## Data Availability

For more details on the access procedure, see the Taiwan Biobank website: https://www.twbiobank.org.tw/

## Article highlights

- Metabolic syndrome (MetS) is a cluster of conditions involving six traits. Approximately 40% of 60-year-old U.S. adults and 37% of Taiwanese adults aged older than 40 years have MetS, causing a substantial economic burden
- This work analyzed the Taiwan Biobank data and estimated 196 (4×7×7=196) mediation effects (four smoking exposures; seven DNA methylation markers covering three generations of epigenetic clocks as mediators; and seven metabolic outcomes).
- GrimEAA and DunedinPACE respectively mediated 48.2% and 24.2% of current smoking’s effect on MetS risk; 60.9% and 26.1% of current smoking pack-years effect on MetS risk; 21.7% and 9.4% of former smoking’s impact on MetS risk; 30.4% and 13.0% of former smoking pack-years effect on MetS risk.
- The GrimEAA-mediated proportions were approximately two times greater than the DunedinPACE-mediated proportions. One component of GrimEAA, DNAmPAI1, mediated the effect of current smoking on all seven metabolic outcomes.
