# Supplementary material for: Three generations of epigenetic clocks in mediating the adverse effect of smoking on metabolic health
Source: Epigenomics. 2025 Apr 18;17(7):453–61. doi: 10.1080/17501911.2025.2494497 (PMC12026080; doi:10.1080/17501911.2025.2494497)
Supplement: Supplemental Material [file IEPI_A_2494497_SM6600.docx]

**Supplementary Materials**

**Three generations of epigenetic clocks in mediating the adverse effect of smoking on metabolic health**

Contents

[Supplementary Method 3](#_Toc195123198)

[Details of Statistical Analyses 3](#_Toc195123199)

[Table S1. The relationship between smoking intensity and the percentage of metabolic syndrome 5](#_Toc195123200)

[Table S2. Total effect of current smoking status on seven metabolic outcomes and the indirect effect associated with seven DNAm markers 6](#_Toc195123201)

[Table S3. Total effect of current smoking pack-years on seven metabolic outcomes and the indirect effect associated with seven DNAm markers 8](#_Toc195123202)

[Table S4. Total effect of former smoking status on seven metabolic outcomes and the indirect effect associated with seven DNAm markers 10](#_Toc195123203)

[Table S5. Total effect of former smoking pack-years on seven metabolic outcomes and the indirect effect associated with seven DNAm markers 12](#_Toc195123204)

[Supplementary references 14](#_Toc195123205)

# Supplementary Method

# Details of Statistical Analyses

The 'mediation' R package [1] fitted two models as follows,

$M (epigenetic marker)=\beta_{01}+\beta_{C1}CS+\beta_{F1}FS+\boldsymbol{\beta}_{\boldsymbol{Z}\boldsymbol{1}}^{\boldsymbol{'}}\boldsymbol{Covariates}+\varepsilon_{1}$; (1)

$Y (trait)=\beta_{02}+\beta_{C2}CS+\beta_{F2}FS+\beta_{M}M+\boldsymbol{\beta}_{\boldsymbol{Z}\boldsymbol{2}}^{\boldsymbol{'}}\boldsymbol{Covariates}+\varepsilon_{2}$. (2)

*CS* and *FS* are indicator variables coding current smoking status (1: yes vs. 0: no) and former smoking status (1: yes vs. 0: no), respectively. $\boldsymbol{Covariates}$ is a vector incorporating chronological age, sex, educational attainment, BMI, performing regular exercise, drinking status, and the proportions of five cell types (B lymphocytes, CD4+ T cells, CD8+ T cells, monocytes, and natural killer cells) estimated by the Houseman deconvolution method [2]. $\varepsilon_{1}$ and $\varepsilon_{2}$ are random error terms of the models (1) & (2). The direct effect of current smoking on the trait is $\hat{\beta_{C2}}$, whereas the indirect (or mediation) effect of current smoking on the trait is $\hat{\beta_{C1}}\times\hat{\beta_{M}}$. The total effect is the sum of direct effect and indirect (or mediation) effect, i.e., $\hat{\beta_{C2}}+\hat{\beta_{C1}}\times\hat{\beta_{M}}$.

Similarly, the direct effect of former smoking on the trait is $\hat{\beta_{F2}}$, whereas the indirect (or mediation) effect of current smoking on the trait is $\hat{\beta_{F1}}\times\hat{\beta_{M}}$. The total effect is the sum of direct effect and indirect (or mediation) effect, i.e., $\hat{\beta_{F2}}+\hat{\beta_{F1}}\times\hat{\beta_{M}}$. When investigating the pack-year model, *CS* and *FS* in models (1) & (2) are changed to the pack-years of current and former smokers, respectively. Investigators may want to combine the information on smoking pack-years from former and current smokers into a single variable representing overall smoking pack-years and use that variable in the models. However, the effect of former smokers’ pack-years and that of current smokers’ pack-years may be different. For example, five pack-years counted from current smokers may significantly impact current DNAm levels more than five pack-years from former smokers.

When the outcome (*Y*) is binary, such as MetS (yes vs. no), we used the “logit” link function to connect the response variable with the explanatory variables [1]. Therefore, model (2) is revised as follows,

$log\frac{\mathrm{Prob}\left( Y=1 \right)}{1-\mathrm{Prob}\left( Y=1 \right)}=\beta_{02}+\beta_{C2}CS+\beta_{F2}FS+\beta_{M}M+\boldsymbol{\beta}_{\boldsymbol{Z}\boldsymbol{2}}^{\boldsymbol{'}}\boldsymbol{Covariates}$, (3)

where $\mathrm{Prob}\left( Y=1 \right)=\frac{exp\left\{ \beta_{02}+\beta_{C2}CS+\beta_{F2}FS+\beta_{M}M+\boldsymbol{\beta}_{\boldsymbol{Z}\boldsymbol{2}}^{\boldsymbol{'}}\boldsymbol{Covariates} \right\}}{1+exp\left\{ \beta_{02}+\beta_{C2}CS+\beta_{F2}FS+\beta_{M}M+\boldsymbol{\beta}_{\boldsymbol{Z}\boldsymbol{2}}^{\boldsymbol{'}}\boldsymbol{Covariates} \right\}}$ is the probability of developing MetS. Take current smoking (*CS*) as an example. The direct effect of *CS* is the average of ${DE}_{\mathrm{treated}}$ and ${DE}_{\mathrm{control}}$, where

${DE}_{\mathrm{treated}}$ = $\frac{1}{2474}\sum_{i=1}^{2474} \left( \mathrm{Prob}\left\{ Y_{i}=1|{CS}_{i}=1, M\left( {CS}_{i}=1 \right) \right\}-\mathrm{Prob}\left\{ Y_{i}=1|{CS}_{i}=0, M\left( {CS}_{i}=1 \right) \right\} \right)$ and

${DE}_{\mathrm{control}}$ =$\frac{1}{2474}\sum_{i=1}^{2474} \left( \mathrm{Prob}\left\{ Y_{i}=1|{CS}_{i}=1, M\left( {CS}_{i}=0 \right) \right\}-\mathrm{Prob}\left\{ Y_{i}=1|{CS}_{i}=0, M\left( {CS}_{i}=0 \right) \right\} \right)$. (4)

$M\left( CS=1 \right)$ and $M\left( CS=0 \right)$ are the predicted epigenetic markers given *CS* = 1 and *CS* = 0, respectively, based on model (1). ${DE}_{\mathrm{treated}}$ is the direct effect of *CS* while fixing the predicted epigenetic marker from the treated group (i.e., *CS* = 1). ${DE}_{\mathrm{control}}$ is the direct effect of *CS* while fixing the predicted epigenetic marker from the control group (i.e., *CS* = 0). In this way, the 'mediation' R package [1] extracts the direct effect from the total effect.

The indirect effect of *M* is the average of $\mathrm{IE}_{\mathrm{treated}}$ and $\mathrm{IE}_{\mathrm{control}}$, where

$\mathrm{IE}_{\mathrm{treated}}$ = $\frac{1}{2474}\sum_{i=1}^{2474} \left( \mathrm{Prob}\left\{ Y_{i}=1|{CS}_{i}=1, M\left( {CS}_{i}=1 \right) \right\}-\mathrm{Prob}\left\{ Y_{i}=1|{CS}_{i}=1, M\left( {CS}_{i}=0 \right) \right\} \right)$ and

$\mathrm{IE}_{\mathrm{control}}$ = $\frac{1}{2474}\sum_{i=1}^{2474} \left( \mathrm{Prob}\left\{ Y_{i}=1|{CS}_{i}=0, M\left( {CS}_{i}=1 \right) \right\}-\mathrm{Prob}\left\{ Y_{i}=1|{CS}_{i}=0, M\left( {CS}_{i}=0 \right) \right\} \right)$. (5)

$\mathrm{IE}_{\mathrm{treated}}$ is the indirect effect of *CS* through the epigenetic marker for the treated group (i.e., *CS* = 1). $\mathrm{IE}_{\mathrm{control}}$ is the indirect effect of *CS* through the epigenetic marker for the control group (i.e., *CS* = 0).

Summing the direct and indirect effects, we have the total effect as follows,

$$\frac{1}{2}\left\{ {DE}_{\mathrm{treated}}+{DE}_{\mathrm{control}} \right\}+\frac{1}{2}\left\{ \mathrm{IE}_{\mathrm{treated}}+\mathrm{IE}_{\mathrm{control}} \right\}$$

$= \frac{1}{2474}\sum_{i=1}^{2474} \left( \mathrm{Prob}\left\{ Y_{i}=1|{CS}_{i}=1, M\left( {CS}_{i}=1 \right) \right\}-\mathrm{Prob}\left\{ Y_{i}=1|{CS}_{i}=0, M\left( {CS}_{i}=0 \right) \right\} \right)$ (6)

# Table S1. The relationship between smoking intensity and the percentage of metabolic syndrome

|  | Former smokers | | Current smokers | |
| --- | --- | --- | --- | --- |
| Number of cigarettes per day | Number of individuals | Percentage of metabolic syndrome | Number of individuals | Percentage of metabolic syndrome |
| 1~10 | 131 | 19.8 % | 152 | 27.6 % |
| 11~20 | 106 | 29.2 % | 99 | 32.3 % |
| >20 | 75 | 33.3 % | 32 | 43.8 % |
| Total | 312 |  | 283 |  |

# Table S2. Total effect of current smoking status on seven metabolic outcomes and the indirect effect associated with seven DNAm markers

| Outcome ^a^ | Total effect | | Unit | 95% Confidence interval | | | | | | *p*-value | | Sample size | |  |
| --- | --- | --- | --- | --- | --- | --- | --- | --- | --- | --- | --- | --- | --- | --- |
| Waist circumference | 0.0465 | | sd | -0.0216 | | | 0.1147 | | | 0.1806 | | 2469 | |  |
| SBP | -0.0155 | | sd | -0.1323 | | | 0.1012 | | | 0.7942 | | 2469 | |  |
| DBP | -0.0884 | | sd | -0.2076 | | | 0.0309 | | | 0.1463 | | 2469 | |  |
| FG | **0.2529** | | sd | **0.1210** | | | **0.3848** | | | **< 0.001** | | 2469 | |  |
| Triglyceride | **0.2304** | | sd | **0.1005** | | | **0.3603** | | | **< 0.001** | | 2469 | |  |
| HDL-C | **-0.3489** | | sd | **-0.4660** | | | **-0.2319** | | | **< 0.001** | | 2469 | |  |
| MetS | **0.0993** | | prob. | **0.0474** | | | **0.1535** | | | **< 0.001** | | 2469 | |  |
| Outcome | Mediator | Unit | | | Mediation effect ^b^ | 95% Confidence interval | | | FDR ^c^ | | Proportion mediated ^d^ (%) | | Sample size | |
| Waist circumference | HannumEAA | sd | | | 0.0024 | -0.0052 | | 0.0100 | 0.6282 | | 5.2 | | 2462 | |
| SBP |  | sd | | | 0.0095 | -0.0029 | | 0.0243 | 0.2105 | | -61.3 | | 2462 | |
| DBP |  | sd | | | 0.0126 | 0.0003 | | 0.0270 | 0.1015 | | -14.3 | | 2462 | |
| FG |  | sd | | | 0.0119 | 5.0E-05 | | 0.0268 | 0.1057 | | 4.7 | | 2462 | |
| Triglyceride |  | sd | | | 0.0107 | -0.0026 | | 0.0263 | 0.1820 | | 4.6 | | 2462 | |
| HDL-C |  | sd | | | -0.0138 | -0.0282 | | -0.0014 | 0.0817 | | 4.0 | | 2462 | |
| MetS |  | prob. | | | 0.0033 | -0.0023 | | 0.0093 | 0.3347 | | 3.3 | | 2462 | |
| Waist circumference | IEAA | sd | | | -0.0005 | -0.0035 | | 0.0020 | 0.7708 | | -1.1 | | 2468 | |
| SBP |  | sd | | | 0.0030 | -0.0013 | | 0.0103 | 0.3161 | | -19.4 | | 2468 | |
| DBP |  | sd | | | 0.0058 | -0.0021 | | 0.0152 | 0.2377 | | -6.6 | | 2468 | |
| FG |  | sd | | | -6.0E-05 | -0.0051 | | 0.0045 | 0.9891 | | 0.0 | | 2468 | |
| Triglyceride |  | sd | | | 0.0011 | -0.0031 | | 0.0067 | 0.7026 | | 0.5 | | 2468 | |
| HDL-C |  | sd | | | -0.0016 | -0.0075 | | 0.0020 | 0.5632 | | 0.5 | | 2468 | |
| MetS |  | prob. | | | -1.0E-05 | -0.0022 | | 0.0021 | 0.9950 | | 0.0 | | 2468 | |
| Waist circumference | PhenoEAA | sd | | | 0.0025 | -0.0066 | | 0.0115 | 0.6801 | | 5.4 | | 2467 | |
| SBP |  | sd | | | 0.0178 | 0.0017 | | 0.0370 | 0.0889 | | -114.8 | | 2467 | |
| DBP |  | sd | | | **0.0221** | **0.0062** | | **0.0415** | **0.0334** | | -25.0 | | 2467 | |
| FG |  | sd | | | 0.0155 | -0.0002 | | 0.0331 | 0.1172 | | 6.1 | | 2467 | |
| Triglyceride |  | sd | | | 0.0036 | -0.0133 | | 0.0190 | 0.7280 | | 1.6 | | 2467 | |
| HDL-C |  | sd | | | -0.0129 | -0.0287 | | 0.0020 | 0.1738 | | 3.7 | | 2467 | |
| MetS |  | prob. | | | 0.0043 | -0.0030 | | 0.0122 | 0.3161 | | 4.3 | | 2467 | |
| Waist circumference | GrimEAA | sd | | | 0.0396 | -0.0012 | | 0.0789 | 0.1251 | | 85.2 | | 2464 | |
| SBP |  | sd | | | 0.0575 | -0.0095 | | 0.1277 | 0.1715 | | -371.0 | | 2464 | |
| DBP |  | sd | | | 0.0468 | -0.0248 | | 0.1202 | 0.2954 | | -52.9 | | 2464 | |
| FG |  | sd | | | **0.2421** | **0.1399** | | **0.3540** | **< 0.001** | | 95.7 | | 2464 | |
| Triglyceride |  | sd | | | **0.1805** | **0.1101** | | **0.2586** | **< 0.001** | | 78.3 | | 2464 | |
| HDL-C |  | sd | | | **-0.1506** | **-0.2138** | | **-0.0840** | **< 0.001** | | 43.2 | | 2464 | |
| MetS |  | prob. | | | **0.0479** | **0.0181** | | **0.0783** | **< 0.001** | | 48.2 | | 2464 | |
| Waist circumference | DNAmPACKYRS | sd | | | 0.0345 | -0.0218 | | 0.0932 | 0.3161 | | 74.2 | | 2415 | |
| SBP |  | sd | | | -0.0032 | -0.1058 | | 0.0952 | 0.9800 | | 20.6 | | 2415 | |
| DBP |  | sd | | | -0.0431 | -0.1488 | | 0.0598 | 0.5432 | | 48.8 | | 2415 | |
| FG |  | sd | | | 0.1515 | 0.0260 | | 0.2868 | 0.0608 | | 59.9 | | 2415 | |
| Triglyceride |  | sd | | | 0.1232 | 0.0228 | | 0.2307 | 0.0653 | | 53.5 | | 2415 | |
| HDL-C |  | sd | | | -0.1156 | -0.2111 | | -0.0219 | 0.0608 | | 33.1 | | 2415 | |
| MetS |  | prob. | | | 0.0357 | -0.0084 | | 0.0799 | 0.1821 | | 36.0 | | 2415 | |
| Waist circumference | DNAmPAI1 | sd | | | **0.0135** | **0.0031** | | **0.0251** | **0.0490** | | 29.0 | | 2469 | |
| SBP |  | sd | | | **0.0276** | **0.0105** | | **0.0479** | **< 0.001** | | -178.1 | | 2469 | |
| DBP |  | sd | | | **0.0259** | **0.0095** | | **0.0447** | **< 0.001** | | -29.3 | | 2469 | |
| FG |  | sd | | | **0.0979** | **0.0623** | | **0.1409** | **< 0.001** | | 38.7 | | 2469 | |
| Triglyceride |  | sd | | | **0.0915** | **0.0591** | | **0.1313** | **< 0.001** | | 39.7 | | 2469 | |
| HDL-C |  | sd | | | **-0.0568** | **-0.0797** | | **-0.0366** | **< 0.001** | | 16.3 | | 2469 | |
| MetS |  | prob. | | | **0.0337** | **0.0223** | | **0.0460** | **< 0.001** | | 33.9 | | 2469 | |
| Waist circumference | DunedinPACE | sd | | | 0.0097 | -0.0081 | | 0.0282 | 0.3959 | | 20.9 | | 2468 | |
| SBP |  | sd | | | 0.0411 | 0.0082 | | 0.0768 | 0.0653 | | -265.2 | | 2468 | |
| DBP |  | sd | | | 0.0282 | -0.0026 | | 0.0631 | 0.1650 | | -31.9 | | 2468 | |
| FG |  | sd | | | **0.0589** | **0.0163** | | **0.1041** | **0.0053** | | 23.3 | | 2468 | |
| Triglyceride |  | sd | | | **0.0831** | **0.0509** | | **0.1187** | **< 0.001** | | 36.1 | | 2468 | |
| HDL-C |  | sd | | | **-0.1143** | **-0.1511** | | **-0.0807** | **< 0.001** | | 32.8 | | 2468 | |
| MetS |  | prob. | | | **0.0240** | **0.0106** | | **0.0394** | **< 0.001** | | 24.2 | | 2468 | |

SBP: Systolic blood pressure; DBP: Diastolic blood pressure; FG: Fasting glucose; HDL-C: high-density lipoprotein cholesterol; MetS: Metabolic syndrome.

^a^ Before mediation analysis, we performed z-score transformation on the waist circumference, SBP, DBP, FG level, triglyceride level, and HDL-C level. Therefore, the unit of the total effect and mediation effect is the standard deviation (sd) of the trait.

^b^ Mediation effects refer to the effect of current smoking on seven metabolic outcomes through the mediators (seven epigenetic markers).

^c^ False discovery rate (FDR) was p-value adjusted for multiple testing via the Benjamini-Hochberg approach [27]. FDRs < 0.05 are highlighted in bold.

^d^ Proportion mediated = mediation effect / total effect.

# Table S3. Total effect of current smoking pack-years on seven metabolic outcomes and the indirect effect associated with seven DNAm markers

| Outcome ^a^ | | Total effect | Unit | 95% Confidence interval | | | | | *p*-value | | Sample size | |  |
| --- | --- | --- | --- | --- | --- | --- | --- | --- | --- | --- | --- | --- | --- |
| Waist circumference | | **0.0022** | sd | **4.6E-06** | | **0.0045** | | | **0.0495** | | 2452 | |  |
| SBP | | -0.0005 | sd | -0.0044 | | 0.0033 | | | 0.7877 | | 2452 | |  |
| DBP | | -0.0033 | sd | -0.0072 | | 0.0006 | | | 0.0996 | | 2452 | |  |
| FG | | **0.0125** | sd | **0.0082** | | **0.0168** | | | **< 0.001** | | 2452 | |  |
| Triglyceride | | **0.0098** | sd | **0.0055** | | **0.0140** | | | **< 0.001** | | 2452 | |  |
| HDL-C | | **-0.0088** | sd | **-0.0127** | | **-0.0049** | | | **< 0.001** | | 2452 | |  |
| MetS | | **0.0023** | prob. | **0.0010** | | **0.0037** | | | **< 0.001** | | 2452 | |  |
| Outcome | Mediator | | Unit | Mediation effect ^b^ | 95% Confidence interval | | | FDR ^c^ | | Proportion mediated ^d^ (%) | | Sample size | |
| Waist circumference | HannumEAA | | sd | 5.00E-05 | -0.0001 | | 0.0003 | 0.6606 | | 2.3 | | 2445 | |
| SBP |  | | sd | 0.0002 | -8.0E-05 | | 0.0006 | 0.2533 | | -40.0 | | 2445 | |
| DBP |  | | sd | 0.0003 | 0 | | 0.0007 | 0.1067 | | -9.1 | | 2445 | |
| FG |  | | sd | 0.0003 | -2.0E-05 | | 0.0007 | 0.1425 | | 2.4 | | 2445 | |
| Triglyceride |  | | sd | 0.0002 | -3.0E-05 | | 6.0E-04 | 0.1713 | | 2.0 | | 2445 | |
| HDL-C |  | | sd | -0.0004 | -0.0008 | | -6.0E-05 | 0.0608 | | 4.5 | | 2445 | |
| MetS |  | | prob. | 7.0E-05 | -5.0E-05 | | 0.0002 | 0.3088 | | 3.0 | | 2445 | |
| Waist circumference | IEAA | | sd | 0 | -8.0E-05 | | 5.0E-05 | 0.9353 | | 0.0 | | 2451 | |
| SBP |  | | sd | 3.0E-05 | -0.0001 | | 0.0002 | 0.7928 | | -6.0 | | 2451 | |
| DBP |  | | sd | 5.0E-05 | -2.0E-04 | | 0.0003 | 0.7708 | | -1.5 | | 2451 | |
| FG |  | | sd | 0 | -1.0E-04 | | 8.0E-05 | 0.9790 | | 0.0 | | 2451 | |
| Triglyceride |  | | sd | 1.0E-05 | -8.0E-05 | | 0.0002 | 0.8465 | | 0.1 | | 2451 | |
| HDL-C |  | | sd | -2.0E-05 | -0.0002 | | 8.0E-05 | 0.8493 | | 0.2 | | 2451 | |
| MetS |  | | prob. | 0 | -4.0E-05 | | 3.0E-05 | 0.9705 | | 0.0 | | 2451 | |
| Waist circumference | PhenoEAA | | sd | 5.0E-05 | -0.0002 | | 0.0003 | 0.7190 | | 2.3 | | 2450 | |
| SBP |  | | sd | 0.0004 | 3.0E-05 | | 0.0009 | 0.0859 | | -80.0 | | 2450 | |
| DBP |  | | sd | **0.0005** | **0.0001** | | **0.0009** | **0.0470** | | -15.2 | | 2450 | |
| FG |  | | sd | 0.0003 | -3.0E-05 | | 0.0008 | 0.1388 | | 2.4 | | 2450 | |
| Triglyceride |  | | sd | 8.0E-05 | -3.0E-04 | | 0.0005 | 0.7280 | | 0.8 | | 2450 | |
| HDL-C |  | | sd | -0.0003 | -0.0007 | | -1.0E-05 | 0.1015 | | 3.4 | | 2450 | |
| MetS |  | | prob. | 9.0E-05 | -5.0E-05 | | 0.0002 | 0.3088 | | 3.9 | | 2450 | |
| Waist circumference | GrimEAA | | sd | 0.0009 | -0.0003 | | 0.0021 | 0.2105 | | 40.9 | | 2448 | |
| SBP |  | | sd | 0.0016 | -0.0006 | | 0.0038 | 0.2377 | | -320.0 | | 2448 | |
| DBP |  | | sd | 0.0014 | -0.0008 | | 0.0036 | 0.2954 | | -42.4 | | 2448 | |
| FG |  | | sd | **0.0062** | **0.0035** | | **0.0096** | **< 0.001** | | 49.6 | | 2448 | |
| Triglyceride |  | | sd | **0.0052** | **0.0032** | | **0.0072** | **< 0.001** | | 53.1 | | 2448 | |
| HDL-C |  | | sd | **-0.0056** | **-0.0080** | | **-0.0036** | **< 0.001** | | 63.6 | | 2448 | |
| MetS |  | | prob. | **0.0014** | **0.0006** | | **0.0023** | **< 0.001** | | 60.9 | | 2448 | |
| Waist circumference | DNAmPACKYRS | | sd | 0.0016 | -0.0004 | | 0.0037 | 0.2105 | | 72.7 | | 2399 | |
| SBP |  | | sd | 0.0001 | -0.0034 | | 0.0038 | 0.9790 | | -20.0 | | 2399 | |
| DBP |  | | sd | -0.0013 | -0.0058 | | 0.0025 | 0.6101 | | 39.4 | | 2399 | |
| FG |  | | sd | 0.0038 | -0.0010 | | 0.0089 | 0.1780 | | 30.4 | | 2399 | |
| Triglyceride |  | | sd | 0.0041 | 0.0002 | | 0.0085 | 0.0980 | | 41.8 | | 2399 | |
| HDL-C |  | | sd | **-0.0060** | **-0.0097** | | **-0.0025** | **0.0140** | | 68.2 | | 2399 | |
| MetS |  | | prob. | 0.0015 | -1.0E-05 | | 0.0029 | 0.1120 | | 65.2 | | 2399 | |
| Waist circumference | DNAmPAI1 | | sd | **0.0004** | **1.0E-04** | | **0.0008** | **0.0261** | | 18.2 | | 2452 | |
| SBP |  | | sd | **0.0008** | **3.0E-04** | | **0.0015** | **0.0098** | | -160.0 | | 2452 | |
| DBP |  | | sd | **0.0008** | **0.0003** | | **0.0014** | **0.0182** | | -24.2 | | 2452 | |
| FG |  | | sd | **0.0027** | **0.0016** | | **0.0042** | **< 0.001** | | 21.6 | | 2452 | |
| Triglyceride |  | | sd | **0.0026** | **0.0015** | | **0.0039** | **< 0.001** | | 26.5 | | 2452 | |
| HDL-C |  | | sd | **-0.0017** | **-0.0026** | | **-0.0010** | **< 0.001** | | 19.3 | | 2452 | |
| MetS |  | | prob. | **0.0009** | **0.0005** | | **0.0013** | **< 0.001** | | 39.1 | | 2452 | |
| Waist circumference | DunedinPACE | | sd | 0.0002 | -3.0E-04 | | 0.0007 | 0.5528 | | 9.1 | | 2451 | |
| SBP |  | | sd | 0.0012 | 0.0002 | | 0.0023 | 0.0544 | | -240.0 | | 2451 | |
| DBP |  | | sd | 0.0008 | -0.0001 | | 0.0019 | 0.1740 | | -24.2 | | 2451 | |
| FG |  | | sd | **0.0015** | **0.0004** | | **0.0027** | **0.0490** | | 12.0 | | 2451 | |
| Triglyceride |  | | sd | **0.0023** | **0.0014** | | **0.0034** | **< 0.001** | | 23.5 | | 2451 | |
| HDL-C |  | | sd | **-0.0035** | **-0.0046** | | **-0.0025** | **< 0.001** | | 39.8 | | 2451 | |
| MetS |  | | prob. | **0.0006** | **0.0003** | | **0.0010** | **< 0.001** | | 26.1 | | 2451 | |

SBP: Systolic blood pressure; DBP: Diastolic blood pressure; FG: Fasting glucose; HDL-C: high-density lipoprotein cholesterol; MetS: Metabolic syndrome.

^a^ Before mediation analysis, we performed z-score transformation on the waist circumference, SBP, DBP, FG level, triglyceride level, and HDL-C level. Therefore, the unit of the total effect and mediation effect is the standard deviation (sd) of the trait.

^b^ Mediation effects refer to the effect of current smoking pack-years on seven metabolic outcomes through the mediators (seven epigenetic markers).

^c^ False discovery rate (FDR) was p-value adjusted for multiple testing via the Benjamini-Hochberg approach [27]. FDRs < 0.05 are highlighted in bold.

^d^ Proportion mediated = mediation effect / total effect.

# Table S4. Total effect of former smoking status on seven metabolic outcomes and the indirect effect associated with seven DNAm markers

| Outcome ^a^ | Total effect | Unit | | 95% Confidence interval | | | | | | *p*-value | | Sample size | |  |
| --- | --- | --- | --- | --- | --- | --- | --- | --- | --- | --- | --- | --- | --- | --- |
| Waist circumference | **0.1013** | sd | | **0.0377** | | | **0.1650** | | | **0.0018** | | 2469 | |  |
| SBP | 0.0648 | sd | | -0.0443 | | | 0.1739 | | | 0.2441 | | 2469 | |  |
| DBP | 0.0660 | sd | | -0.0454 | | | 0.1774 | | | 0.2454 | | 2469 | |  |
| FG | 0.0343 | sd | | -0.0888 | | | 0.1575 | | | 0.5846 | | 2469 | |  |
| Triglyceride | -0.0569 | sd | | -0.1783 | | | 0.0644 | | | 0.3577 | | 2469 | |  |
| HDL-C | -0.0840 | sd | | -0.1933 | | | 0.0254 | | | 0.1322 | | 2469 | |  |
| MetS | **0.0534** | prob. | | **0.0087** | | | **0.0992** | | | **0.0193** | | 2469 | |  |
| Outcome | Mediator | | Unit | | Mediation effect ^b^ | 95% Confidence interval | | | FDR ^c^ | | Proportion mediated ^d^ (%) | | Sample size | |
| Waist circumference | HannumEAA | | sd | | 0.0008 | -0.002 | | 0.0043 | 0.6776 | | 0.8 | | 2462 | |
| SBP |  | | sd | | 0.0034 | -0.002 | | 0.0108 | 0.3221 | | 5.2 | | 2462 | |
| DBP |  | | sd | | 0.0045 | -0.001 | | 0.0119 | 0.1800 | | 6.8 | | 2462 | |
| FG |  | | sd | | 0.0042 | -0.001 | | 0.0119 | 0.1976 | | 12.2 | | 2462 | |
| Triglyceride |  | | sd | | 0.0038 | -0.001 | | 0.0117 | 0.2431 | | -6.7 | | 2462 | |
| HDL-C |  | | sd | | -0.0049 | -0.013 | | 0.0005 | 0.1738 | | 5.8 | | 2462 | |
| MetS |  | | prob. | | 0.0011 | -0.001 | | 0.0039 | 0.3470 | | 2.1 | | 2462 | |
| Waist circumference | IEAA | | sd | | -0.0003 | -0.003 | | 0.0014 | 0.8306 | | -0.3 | | 2468 | |
| SBP |  | | sd | | 0.0017 | -0.002 | | 0.0080 | 0.5618 | | 2.6 | | 2468 | |
| DBP |  | | sd | | 0.0034 | -0.005 | | 0.0127 | 0.4939 | | 5.2 | | 2468 | |
| FG |  | | sd | | -4.0E-05 | -0.004 | | 0.0034 | 0.9790 | | -0.1 | | 2468 | |
| Triglyceride |  | | sd | | 0.0006 | -0.003 | | 0.0049 | 0.8232 | | -1.1 | | 2468 | |
| HDL-C |  | | sd | | -0.0009 | -0.005 | | 0.0020 | 0.6802 | | 1.1 | | 2468 | |
| MetS |  | | prob. | | 0.0000 | -0.002 | | 0.0015 | 0.9911 | | 0.0 | | 2468 | |
| Waist circumference | PhenoEAA | | sd | | 0.0013 | -0.003 | | 0.0065 | 0.6819 | | 1.3 | | 2467 | |
| SBP |  | | sd | | 0.0093 | 0.001 | | 0.0204 | 0.0795 | | 14.4 | | 2467 | |
| DBP |  | | sd | | **0.0116** | **0.003** | | **0.0243** | **0.0368** | | 17.6 | | 2467 | |
| FG |  | | sd | | 0.0081 | 0.000 | | 0.0193 | 0.1300 | | 23.6 | | 2467 | |
| Triglyceride |  | | sd | | 0.0019 | -0.007 | | 0.0111 | 0.7280 | | -3.3 | | 2467 | |
| HDL-C |  | | sd | | -0.0068 | -0.017 | | 0.0012 | 0.1760 | | 8.1 | | 2467 | |
| MetS |  | | prob. | | 0.0022 | -0.001 | | 0.0062 | 0.3062 | | 4.1 | | 2467 | |
| Waist circumference | GrimEAA | | sd | | 0.0101 | 0.000 | | 0.0211 | 0.1047 | | 10.0 | | 2464 | |
| SBP |  | | sd | | 0.0147 | -0.003 | | 0.0326 | 0.1842 | | 22.7 | | 2464 | |
| DBP |  | | sd | | 0.0120 | -0.006 | | 0.0313 | 0.2678 | | 18.2 | | 2464 | |
| FG |  | | sd | | **0.0619** | **0.035** | | **0.0965** | **< 0.001** | | 180.5 | | 2464 | |
| Triglyceride |  | | sd | | **0.0462** | **0.025** | | **0.0712** | **< 0.001** | | -81.2 | | 2464 | |
| HDL-C |  | | sd | | **-0.0385** | **-0.059** | | **-0.0206** | **< 0.001** | | 45.8 | | 2464 | |
| MetS |  | | prob. | | **0.0116** | **0.004** | | **0.0201** | **< 0.001** | | 21.7 | | 2464 | |
| Waist circumference | DNAmPACKYRS | | sd | | 0.0109 | -0.006 | | 0.0296 | 0.3226 | | 10.8 | | 2415 | |
| SBP |  | | sd | | -0.0010 | -0.031 | | 0.0297 | 0.9790 | | -1.5 | | 2415 | |
| DBP |  | | sd | | -0.0136 | -0.045 | | 0.0213 | 0.5528 | | -20.6 | | 2415 | |
| FG |  | | sd | | 0.0478 | 0.009 | | 0.0896 | 0.0631 | | 139.4 | | 2415 | |
| Triglyceride |  | | sd | | 0.0389 | 0.006 | | 0.0752 | 0.0663 | | -68.4 | | 2415 | |
| HDL-C |  | | sd | | -0.0365 | -0.065 | | -0.0056 | 0.0544 | | 43.5 | | 2415 | |
| MetS |  | | prob. | | 0.0106 | -0.002 | | 0.0247 | 0.1840 | | 19.9 | | 2415 | |
| Waist circumference | DNAmPAI1 | | sd | | 0.0036 | 1.0E-05 | | 0.0085 | 0.1047 | | 3.6 | | 2469 | |
| SBP |  | | sd | | 0.0073 | 0.0002 | | 0.0164 | 0.1015 | | 11.3 | | 2469 | |
| DBP |  | | sd | | 0.0068 | 0.0003 | | 0.0160 | 0.0980 | | 10.3 | | 2469 | |
| FG |  | | sd | | 0.0257 | 0.0017 | | 0.0522 | 0.1026 | | 74.9 | | 2469 | |
| Triglyceride |  | | sd | | 0.0240 | 0.0017 | | 0.0476 | 0.0889 | | -42.2 | | 2469 | |
| HDL-C |  | | sd | | -0.0149 | -0.0304 | | -0.0008 | 0.0980 | | 17.7 | | 2469 | |
| MetS |  | | prob. | | 0.0085 | 0.0007 | | 0.0169 | 0.0795 | | 15.9 | | 2469 | |
| Waist circumference | DunedinPACE | | sd | | 0.0021 | -0.0018 | | 0.0070 | 0.4038 | | 2.1 | | 2468 | |
| SBP |  | | sd | | 0.0091 | 0.0013 | | 0.0200 | 0.0606 | | 14.0 | | 2468 | |
| DBP |  | | sd | | 0.0063 | -0.0012 | | 0.0164 | 0.1842 | | 9.5 | | 2468 | |
| FG |  | | sd | | **0.0130** | **0.0029** | | **0.0268** | **0.0223** | | 37.9 | | 2468 | |
| Triglyceride |  | | sd | | **0.0184** | **0.0060** | | **0.0339** | **0.0098** | | -32.3 | | 2468 | |
| HDL-C |  | | sd | | **-0.0253** | **-0.0445** | | **-0.0090** | **0.0140** | | 30.1 | | 2468 | |
| MetS |  | | prob. | | **0.0050** | **0.0014** | | **0.0101** | **0.0053** | | 9.4 | | 2468 | |

SBP: Systolic blood pressure; DBP: Diastolic blood pressure; FG: Fasting glucose; HDL-C: high-density lipoprotein cholesterol; MetS: Metabolic syndrome.

^a^ Before mediation analysis, we performed z-score transformation on the waist circumference, SBP, DBP, FG level, triglyceride level, and HDL-C level. Therefore, the unit of the total effect and mediation effect is the standard deviation (sd) of the trait.

^b^ Mediation effects refer to the effect of former smoking on seven metabolic outcomes through the mediators (seven epigenetic markers).

^c^ False discovery rate (FDR) was p-value adjusted for multiple testing via the Benjamini-Hochberg approach [27]. FDRs < 0.05 are highlighted in bold.

^d^ Proportion mediated = mediation effect / total effect.

# Table S5. Total effect of former smoking pack-years on seven metabolic outcomes and the indirect effect associated with seven DNAm markers

| Outcome ^a^ | Total effect | | Unit | | 95% Confidence interval | | | | *p*-value | | Sample size | |
| --- | --- | --- | --- | --- | --- | --- | --- | --- | --- | --- | --- | --- |
| Waist circumference | **0.0048** | | sd | | **0.0017** | | **0.0080** | | **0.0028** | | 2452 | |
| SBP | 0.0038 | | sd | | -0.0016 | | 0.0093 | | 0.1660 | | 2452 | |
| DBP | 0.0031 | | sd | | -0.0024 | | 0.0087 | | 0.2696 | | 2452 | |
| FG | 0.0040 | | sd | | -0.0021 | | 0.0101 | | 0.1990 | | 2452 | |
| Triglyceride | -0.0002 | | sd | | -0.0063 | | 0.0058 | | 0.9393 | | 2452 | |
| HDL-C | -0.0013 | | sd | | -0.0068 | | 0.0042 | | 0.6375 | | 2452 | |
| MetS | **0.0023** | | prob. | | **0.0004** | | **0.0041** | | **0.0197** | | 2452 | |
| Outcome | Mediator | Unit | | Mediation effect ^b^ | | 95% Confidence interval | | FDR ^c^ | | Proportion mediated ^d^ (%) | | Sample size |
| Waist circumference | HannumEAA | sd | | 4.0E-05 | | -1.0E-04 | 0.0002 | 0.6626 | | 0.8 | | 2445 |
| SBP |  | sd | | 0.0002 | | -0.0001 | 0.0005 | 0.2954 | | 5.3 | | 2445 |
| DBP |  | sd | | 0.0002 | | 0.0000 | 0.0006 | 0.1859 | | 6.5 | | 2445 |
| FG |  | sd | | 2.0E-04 | | -3.0E-05 | 0.0006 | 0.1960 | | 5.0 | | 2445 |
| Triglyceride |  | sd | | 0.0002 | | -5.0E-05 | 0.0006 | 0.2431 | | -100.0 | | 2445 |
| HDL-C |  | sd | | -0.0003 | | -0.0007 | 2.0E-05 | 0.1414 | | 23.1 | | 2445 |
| MetS |  | prob. | | 6.0E-05 | | -4.0E-05 | 0.0002 | 0.3258 | | 2.6 | | 2445 |
| Waist circumference | IEAA | sd | | -3.0E-05 | | -2.0E-04 | 9.0E-05 | 0.6952 | | -0.6 | | 2451 |
| SBP |  | sd | | 0.0002 | | -6.0E-05 | 0.0005 | 0.2895 | | 5.3 | | 2451 |
| DBP |  | sd | | 0.0003 | | -5.0E-05 | 0.0009 | 0.1713 | | 9.7 | | 2451 |
| FG |  | sd | | 0.0000 | | -0.0002 | 0.0002 | 0.9790 | | 0.0 | | 2451 |
| Triglyceride |  | sd | | 7.0E-05 | | -0.0002 | 0.0004 | 0.6801 | | -35.0 | | 2451 |
| HDL-C |  | sd | | -0.0001 | | -0.0004 | 9.0E-05 | 0.4102 | | 7.7 | | 2451 |
| MetS |  | prob. | | 0.0000 | | -0.0001 | 9.0E-05 | 0.9790 | | 0.0 | | 2451 |
| Waist circumference | PhenoEAA | sd | | 6.0E-05 | | -0.0002 | 0.0003 | 0.6802 | | 1.3 | | 2450 |
| SBP |  | sd | | 0.0005 | | 3.0E-05 | 0.0010 | 0.0903 | | 13.2 | | 2450 |
| DBP |  | sd | | **0.0006** | | **0.0001** | **0.0012** | **0.0400** | | 19.4 | | 2450 |
| FG |  | sd | | 0.0004 | | -4.0E-05 | 0.0009 | 0.1420 | | 10.0 | | 2450 |
| Triglyceride |  | sd | | 9.0E-05 | | -0.0004 | 0.0006 | 0.7473 | | -45.0 | | 2450 |
| HDL-C |  | sd | | -4.0E-04 | | -0.0009 | 1.0E-05 | 0.1180 | | 30.8 | | 2450 |
| MetS |  | prob. | | 1.0E-04 | | -6.0E-05 | 0.0003 | 0.2954 | | 4.3 | | 2450 |
| Waist circumference | GrimEAA | sd | | 0.0004 | | -0.0002 | 0.0011 | 0.2377 | | 8.3 | | 2448 |
| SBP |  | sd | | 0.0008 | | -0.0002 | 0.0019 | 0.2377 | | 21.1 | | 2448 |
| DBP |  | sd | | 0.0007 | | -0.0005 | 0.0019 | 0.3289 | | 22.6 | | 2448 |
| FG |  | sd | | **0.0031** | | **0.0017** | **0.0050** | **< 0.001** | | 77.5 | | 2448 |
| Triglyceride |  | sd | | **0.0026** | | **0.0016** | **0.0039** | **< 0.001** | | -1300.0 | | 2448 |
| HDL-C |  | sd | | **-0.0028** | | **-0.0041** | **-0.0017** | **< 0.001** | | 215.4 | | 2448 |
| MetS |  | prob. | | **0.0007** | | **0.0003** | **0.0012** | **< 0.001** | | 30.4 | | 2448 |
| Waist circumference | DNAmPACKYRS | sd | | 7.0E-04 | | -0.0002 | 0.0016 | 0.2167 | | 14.6 | | 2399 |
| SBP |  | sd | | 6.0E-05 | | -0.0016 | 0.0016 | 0.9790 | | 1.6 | | 2399 |
| DBP |  | sd | | -0.0006 | | -0.0025 | 0.0012 | 0.5929 | | -19.4 | | 2399 |
| FG |  | sd | | 0.0017 | | -0.0003 | 0.0039 | 0.1819 | | 42.5 | | 2399 |
| Triglyceride |  | sd | | 0.0018 | | 8.0E-05 | 0.0037 | 0.0980 | | -900.0 | | 2399 |
| HDL-C |  | sd | | **-0.0027** | | **-0.0045** | **-0.0010** | **0.0053** | | 207.7 | | 2399 |
| MetS |  | prob. | | 0.0007 | | 4.0E-05 | 0.0013 | 0.0980 | | 30.4 | | 2399 |
| Waist circumference | DNAmPAI1 | sd | | 0.0002 | | 1.0E-05 | 4.0E-04 | 0.0967 | | 4.2 | | 2452 |
| SBP |  | sd | | 0.0004 | | 4.0E-05 | 8.0E-04 | 0.0742 | | 10.5 | | 2452 |
| DBP |  | sd | | 0.0003 | | 3.0E-05 | 0.0008 | 0.0663 | | 9.7 | | 2452 |
| FG |  | sd | | 0.0012 | | 0.0001 | 0.0024 | 0.0790 | | 30.0 | | 2452 |
| Triglyceride |  | sd | | 0.0012 | | 0.0002 | 0.0022 | 0.0643 | | -600.0 | | 2452 |
| HDL-C |  | sd | | -0.0008 | | -0.0015 | -7.0E-05 | 0.0801 | | 61.5 | | 2452 |
| MetS |  | prob. | | 0.0004 | | 5.0E-05 | 0.0008 | 0.0801 | | 17.4 | | 2452 |
| Waist circumference | DunedinPACE | sd | | 8.0E-05 | | -0.0001 | 0.0003 | 0.5473 | | 1.7 | | 2451 |
| SBP |  | sd | | 0.0005 | | 5.0E-05 | 0.0010 | 0.0643 | | 13.2 | | 2451 |
| DBP |  | sd | | 0.0003 | | -5.0E-05 | 0.0008 | 0.1650 | | 9.7 | | 2451 |
| FG |  | sd | | **0.0006** | | **0.0001** | **0.0013** | **0.0298** | | 15.0 | | 2451 |
| Triglyceride |  | sd | | **9.0E-04** | | **0.0004** | **0.0016** | **0.0098** | | -450.0 | | 2451 |
| HDL-C |  | sd | | **-0.0014** | | **-0.0023** | **-0.0006** | **0.0053** | | 107.7 | | 2451 |
| MetS |  | prob. | | **0.0003** | | **8.0E-05** | **0.0005** | **< 0.001** | | 13.0 | | 2451 |

SBP: Systolic blood pressure; DBP: Diastolic blood pressure; FG: Fasting glucose; HDL-C: high-density lipoprotein cholesterol; MetS: Metabolic syndrome.

^a^ Before mediation analysis, we performed z-score transformation on the waist circumference, SBP, DBP, FG level, triglyceride level, and HDL-C level. Therefore, the unit of the total effect and mediation effect is the standard deviation (sd) of the trait.

^b^ Mediation effects refer to the effect of former smoking pack-years on seven metabolic outcomes through the mediators (seven epigenetic markers).

^c^ False discovery rate (FDR) was p-value adjusted for multiple testing via the Benjamini-Hochberg approach [27]. FDRs < 0.05 are highlighted in bold.

^d^ Proportion mediated = mediation effect / total effect.

# Supplementary references

1. Tingley D, Yamamoto T, Hirose K et al. mediation: R package for Causal Mediation Analysis, Journal of Statistical Software 2014;59:1-38.

2. Houseman EA, Accomando WP, Koestler DC et al. DNA methylation arrays as surrogate measures of cell mixture distribution, BMC Bioinformatics 2012;13:86.
